# Digital twin-based applications in crop monitoring

**DOI:** 10.1016/j.heliyon.2025.e42137

**Published:** 2025-01-21

**Authors:** Tsega Y. Melesse

**Affiliations:** Faculty of Chemical and Food Engineering, Bahir Dar Institute of Technology, Bahir Dar University, Bahir Dar, Ethiopia

**Keywords:** Digital twin, Modeling approaches, Architecture, Benefits, Crop monitoring

## Abstract

Technological advances in agriculture, particularly the use of digital twins, are having a significant impact on crop management. This article explores the use of digital twins in crop management, focusing on modeling methodologies, roles, implementation architecture, challenges, and prospects. The review identifies various modeling methods for digital twin development in crop monitoring, including physics-based, agent-based, data-driven, hybrid, and spatial models. These models provide up-to-date information on environmental conditions, soil moisture, and other variables affecting crop development and yield. Despite being in its early stages of implementation, digital twin technology is showing signs of progress, suggesting it could be the next step in crop farming's digitalization, increasing visibility and transparency, and improving decision-making processes. The review offers valuable insights and fills research gaps, enabling informed decisions for farmers, policymakers, and service providers to enhance productivity, sustainability, and resilience in modern agriculture.

## 1 Introduction

Agriculture faces challenges such as climate change, resource depletion, population growth, and evolving consumer preferences, which threaten productivity and affect crop yields, livestock health, and food system stability. The use of advanced technologies like precision agriculture, smart irrigation systems, biotechnology, data analytics, artificial intelligence, robotics, and sustainable farming practices can significantly boost agricultural productivity. In this case, precision agriculture technologies optimize inputs, smart irrigation systems improve water efficiency, biotechnology improves crop resilience, and data analytics and AI improve crop management. Crop management is one of the sectors that has benefited from recent innovations in agriculture enabled by technologies such as sensors, the Internet of Things (IoT), artificial intelligence (AI), and big data analytics to optimize agricultural production, improve crop yields, reduce costs, and increase efficiency. The Fourth Industrial Revolution has resulted in the development of cutting-edge technology, such as the digital twin, that can considerably improve crop monitoring and management. With this breakthrough, digital twins have emerged as a promising tool in agriculture for improving such management practices [1].

A digital twin is a digital representation of a physical system that is constantly updated with new data and simulations. It provides in-depth information about crop health, growth circumstances, and environmental factors [2]. Traditional crop monitoring technologies often lack real-time, precise, and comprehensive data. A digital twin combines data from sensors, satellite images, and weather forecasts to provide a comprehensive view of crop conditions, enabling early interventions and increased quality and production. It enables real-time data connection with a specific instance of a system or process, providing simulation capabilities for the product's lifecycle and integrating the entire supply chain [[3], [4], [5], [6], [7]]. In precision farming, the digital twin reflects a plant's developmental stages in real-time or near-real-time, predicting growth and enabling continuous monitoring and control of the plant development process [2,8]. It also allows timely identification of problems in the field and provides recommendations based on weather conditions or expert advice [8,9]. Applications of digital twins in agriculture include predictive modeling for crop yield forecasting, monitoring soil health and fertility, and optimizing irrigation schedules. For instance, a digital twin of a farm can help predict pest outbreaks or diseases by integrating data from climate conditions and crop health metrics, allowing farmers to take preemptive actions to prevent crop losses. Furthermore, digital twins are being used to optimize the growth conditions for specific crop types by adjusting environmental factors like temperature, humidity, and soil moisture in a real-time [[10], [11], [12]].

A digital twin is a digital representation of a physical system that is constantly updated with new data and simulations. It can provide in-depth information about crop health, growth circumstances, and environmental factors [[13], [14], [15], [16], [17]]. Crop monitoring technologies that are traditionally used frequently lack real-time, precise, and comprehensive data. Data from sensors, satellite images, and weather forecasts are combined to produce a comprehensive view of crop conditions, allowing for early interventions and increased quality and production.

The research trend in the use of the digital twin has recently gained the interest of researchers in areas such as manufacturing and services, oil and gas, transportation, food and beverage, agriculture, aerospace, construction, and the energy sector has risen significantly [6,[18], [19], [20], [21], [22], [23]]. However, digital twin technology is still in its early phases in the agriculture industry [1,24,25]. Although the scientific community has reported promising advances in digital twin use in crop management [2,17,26] these have not been fully analyzed and reported in the literature. Much research on the application of digital twins does not directly address crop management. This paper explores various digital twin modeling approaches in crop management, including physics-based, data-driven, hybrid, agent-based, and spatial models. Further, it has examined the various benefits of digital twins in crop management and the general architecture of digital twins, challenges, as well as future opportunities associated with the adoption of digital twins.

A systematic review is required to synthesize current research, identify gaps, and suggest future directions. It aims to answer research questions about the development approaches, their role in crop management, the architecture of digital twins, and the challenges and opportunities associated with their application. The remaining content in this paper is structured as follows: Section 2 describes the review methodology. Section 3 provides a detailed discussion in response to the research questions described in the methodology. Finally, Section 4 provides the conclusions of the work.

## 2 Method

### 2.1 Design and search strategy

**Fig. 1:**
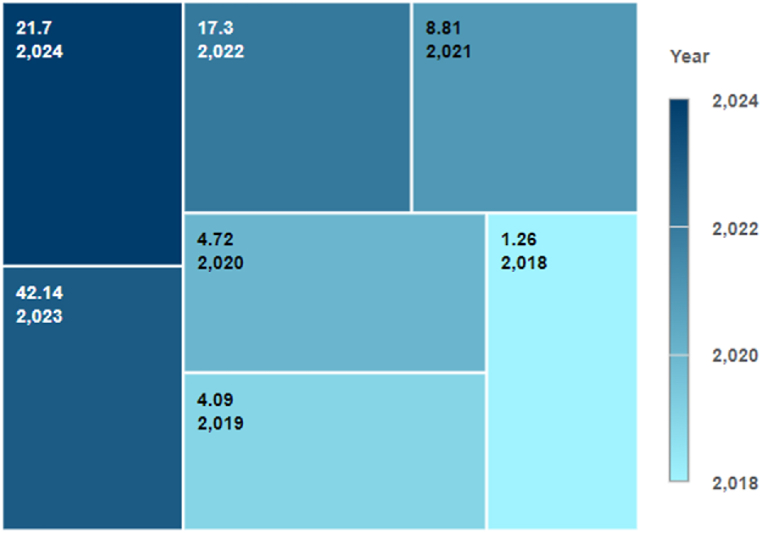
The percentage of publications by year.

The study identified a significant increase in digital twin applications in crop management since 2018, with 1.26 % of the publications published in 2018, 4.09 % in 2019, 4.72 % in 2020, 8.81 % in 2020, 42.14 % in 2023, and 21.7 % in 2024 (Fig. 1).

The search was carried out on Scopus with the article title, abstract, and keywords to identify peer-reviewed papers. Search terms were using the following keywords: (“digital twin” OR “digital twins”) AND (“agriculture” OR “smart farming” OR “precision farming").

**Fig. 2:**
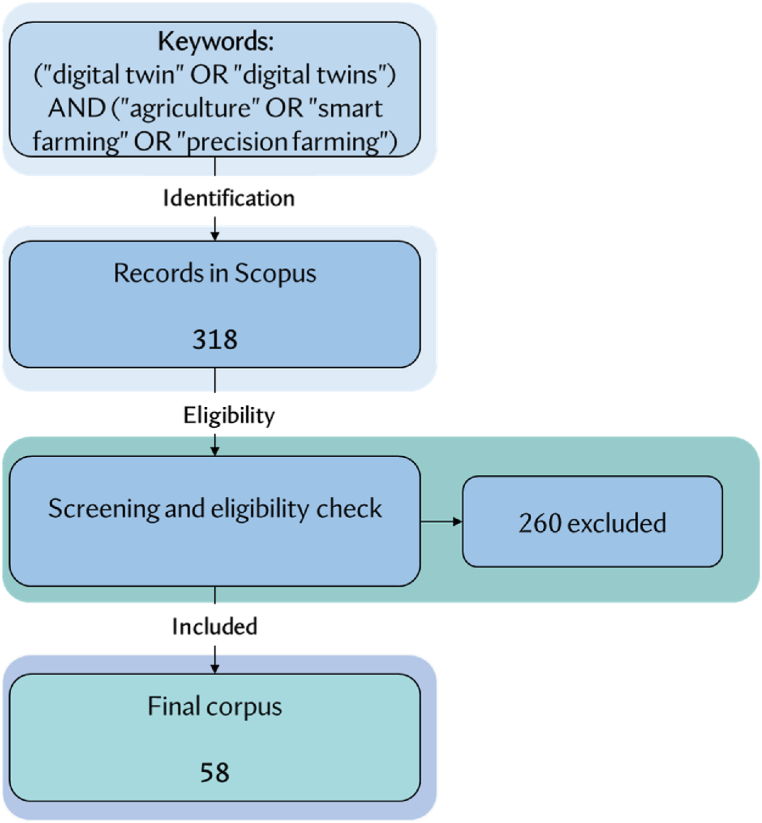
Steps during the literature review.

To investigate how digital twins have been used in the past few years, this study examines published works from 2018 to 2024, following the steps outlined in Fig. 2. In the analysis, only conference papers, articles, review papers, and book chapters were considered.

**Fig. 3:**
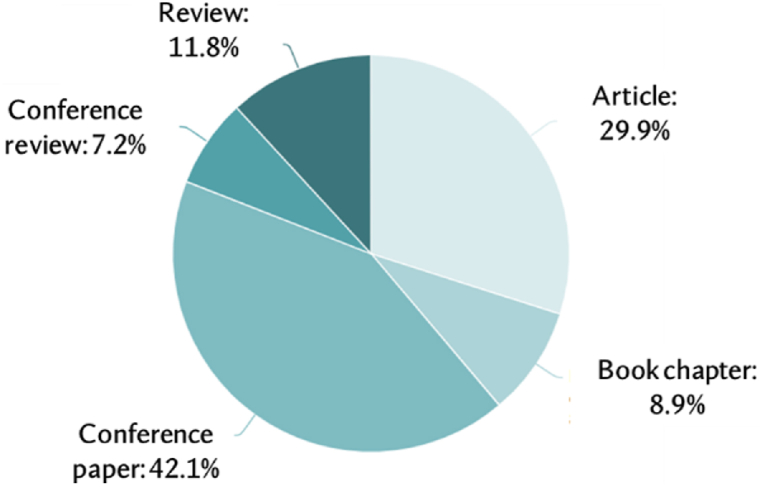
Document type.

Fig. 3 is a pie chart representing the distribution of information in various formats within the topic of study. Conference papers constitute 42.1 % of all published papers, articles 29.9 %, and reviews 11.8 %. Book chapters account for 8.9 %, while conference reviews make up 7.2 %. Overall, conferences represent 49.3 % of all publications.

### 2.2 Screening and eligibility assessment

As shown in Fig. 1, the study identified 318 published papers, with 58 being screened based on title and abstract, full-text evaluations. The final screening and eligibility assessment are done based on predefined assessment criteria described in Table 1.

**Table 1:** Final screening and eligibility assessment.

Screening Exclusion Criteria	Eligibility Exclusion Criteria
Is it a peer-reviewed journal article, a book chapter, a review, or a conference paper?	Is the full document available for reading?
Does the document illustrate the use of digital twins in crop management?	Does the paper answer at least one of the research questions that have already been set?

**Fig. 4:**
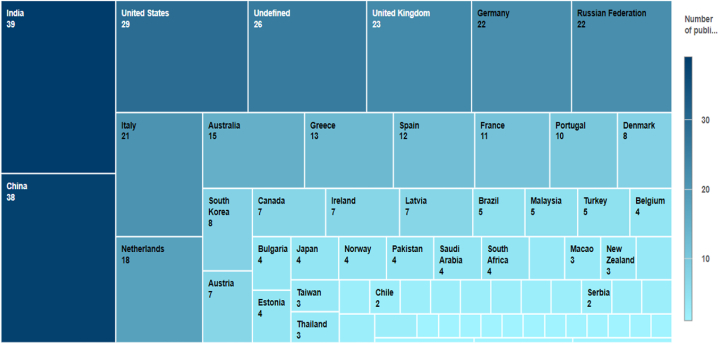
Contribution of countries.

According to the preliminary search results (Fig. 4), India, China, and the United States are the top three contributors to worldwide research publications in the field, with India leading with 39 publications and China behind closely with 38. The United Kingdom has 23 publications, Germany and Russia each have 22, Italy has 21 publications, the Netherlands has 18, Australia has 15, Greece has 13, Spain has 12, France has 11 publications, and Denmark and South Korea have 8 each.

**Fig. 5:**
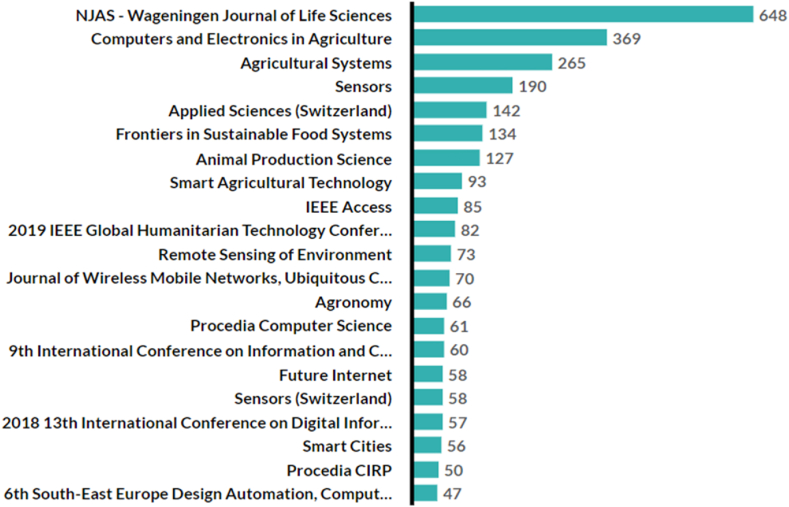
Citation per source title.

As illustrated in Fig. 5, the NJAS-Wageningen Journal of Life Sciences has the highest citation count in life sciences research, particularly digital twin applications. The growing importance of agriculture, technology, and interdisciplinary studies is highlighted by the increasing number of journals and conferences devoted to these fields, with IEEE conferences emphasizing the role of engineering and technology in modern agricultural research.

## 3 Results and discussions

### 3.1 Modeling approaches of the digital twin in crop management

The digital twin can use real-time data, statistical or simulation models, machine learning models, and other techniques that can predict crop behavior in the future [27,28]. However, the accuracy of the predictions is dependent on the adequacy of models used during digital twin development that describe the real processes of plant growth. In this review, five modeling approaches for digital twins are identified and discussed, which include physics-based, agent-based, data-driven, hybrid, and spatial models [16].

Physics-based models simulate crop behavior under various environmental conditions using physical processes like photosynthesis, transpiration, and nutrient uptake [16,29]. Mathematical models use data like dry weight, humidity, temperature, and soil composition to describe crop characteristics [30]. For instance, crop models like CERES (Crop Environment Resource Synthesis) and APSIM (Agricultural Production Systems Simulator) are widely used to simulate crop growth and predict responses to environmental stresses [31]. However, their validity continues to be questioned due to their reliance on simplifications that may not fully capture complex ecological systems [32]. Furthermore, the accuracy of these models is often limited by the quality and resolution of input data, such as soil properties and climate forecasts. In some regions, these models struggle to account for irregular rainfall patterns or extreme climatic events, which can significantly affect crop productivity.

Data-driven models use machine learning algorithms to predict crop yields, detect anomalies, and optimize resource usage [16,33]. Examples of such models include neural networks and random forest algorithms that can predict crop diseases, estimate yields, and monitor soil moisture in real time [34,35]. They outperform physics-based models in terms of accuracy and are simple to implement [36]. Notably, deep learning techniques have been used to analyze satellite images to predict crop yields in regions like India and the United States, showing higher prediction accuracy compared to traditional methods [37,38]. These models are also being employed to detect pests, such as aphids or locusts, by analyzing image data from drone or satellite footage, which enables more proactive pest management strategies [39,40].

Hybrid models combine physical-based and data-driven approaches to create more accurate and flexible models. They can simulate crop growth under various environmental conditions and adapt to changing conditions in a real-time. A specific instance of a hybrid approach is the integration of APSIM with machine learning algorithms to enhance predictions of crop responses to fluctuating weather conditions [41]. Similarly, hybrid models that combine crop modeling with sensor networks in greenhouse environments have been used to optimize conditions for plant growth, taking into account variables such as light, temperature, and humidity [42]. These models benefit from both the physical understanding of crop physiology and the adaptability of data-driven approaches.

Agent-based models simulate the behavior of individual plants or insects in a crop, studying the effects of different management practices on crop growth and pest populations [43]. They can create and maintain digital twins of plants that reflect their conditions and parameters for farm management. For instance, agent-based models have been used to simulate the spread of diseases, such as wheat rust, by modeling the interactions between infected plants and environmental variables [44]. These models can be extended to simulate the behavior of agricultural workers or machinery, as in the case of optimizing planting patterns or irrigation strategies. Multi-agent strategies can be implemented to develop plant digital twins that reflect plant growth phases and enable more accurate production forecasts and agrotechnical planning [10].

Spatial models are built in conjunction with a geographical information system (GIS) to describe basic processes and properties for a given set of spatial features [45,46]. They are critical to sustainable agricultural practices by considering variability in both space and time [47]. According to reports, GIS-based models have been used to assess soil erosion risk in vineyard management, enabling farmers to implement targeted soil conservation practices based on site-specific conditions [48]. Such models have been employed to optimize irrigation schedules and resource distribution in large-scale agricultural fields, taking into account soil type, elevation, and water availability [49,50]**.** These models are also important in land-use planning, where they help identify areas for optimal crop rotation, maximizing yield while minimizing environmental degradation.

The implementation of these modeling methodologies is based on stakeholder demands and crop cultivation. For instance, physical-based models work well for precision agricultural applications, whereas data-driven models are useful for yield prediction and anomaly detection. Hybrid and agent-based models are effective for complex crop systems, while spatial models help optimize resource usage over large areas [51,52]. Hybrid techniques often utilize the expertise of agronomists who have studied plant growth for decades [28]. In the case of hybrid models, the integration of expert knowledge with data-driven predictions ensures that these models remain adaptable to rapidly changing agricultural conditions.

### 3.2 Role of the digital twin in crop monitoring

Digital twin technology enables precise and data-driven decision-making, thereby increasing crop productivity and resource efficiency. By integrating data from various sources, such as weather patterns, soil composition, plant genetics, and growth data, digital twins generate insights and predictions about crop growth and resource utilization on the farm [53]. Integrating data from soil sensors with weather forecasts can help determine the best time for irrigation and fertilization. These insights assist in optimizing the timing and quantity of inputs, reducing wastage, and improving crop yields. Digital twins provide real-time insights into the crop's health, enabling more informed decisions regarding operations like irrigation, fertilization, and pest control [54,55]. In a practical example, a farm using a digital twin for pest management can receive early warnings of pest outbreaks and take preventive action, reducing pesticide use and ensuring crop health. As digital twin technology advances, it is expected to play a growing role in automating tasks like planting, harvesting, and pesticide spraying. For instance, autonomous drones equipped with digital twins could plant seeds and spray fertilizers or pesticides based on real-time environmental data [56]. One of the key benefits of digital twin technology is the ability to collaborate and share data with stakeholders in the agriculture industry.

In irrigation, digital twins can evaluate the behavior of an irrigation system before implementing it in the field and evaluating different irrigation strategies in parallel with current farm practices [25]. By simulating the performance of different irrigation methods (drip, sprinkler, or flood irrigation), digital twins allow farmers to determine the most efficient system tailored to their land's characteristics and local weather patterns. This enhances visibility in farm operations and reduces water usage by allowing farmers to gather soil, weather, and crop information and evaluate multiple irrigation strategies. A practical application can be seen in arid regions where water scarcity is a concern, and farmers can use digital twin systems to optimize irrigation schedules, minimizing water waste.

Digital twins can also model different scenarios, such as the impact of changes in environmental conditions on crop growth, enabling farmers to make informed decisions about crop management in a changing environment [51,57,58]. For example, simulations may show how rising temperatures or drought conditions could affect crop health, allowing farmers to take proactive measures like adjusting planting times or selecting more drought-resistant crops. The use of such technology and applied computing enables local farmers to better control production losses, allowing them to grow crops more sustainably while minimizing their carbon footprint [59]. In addition, these systems can help monitor soil health and recommend optimal crop rotation practices to maintain soil fertility, ultimately leading to improved environmental sustainability.

Table 2 outlines the various applications of digital twins in crop management, including crop health monitoring, farm management, irrigation, pest and disease management, supply chain optimization, yield prediction, climate impact, resource optimization, and sustainability compliance.

**Table 2:** Main application areas of digital twin in agriculture.

Application areas	Role of Digital Twin	References
Monitoring crop health	Enable real-time crop health monitoring	[2,60]
Farm management and planning	Offer a comprehensive view of farm operations	[51,58,61,62]
Irrigation management	Optimize farm irrigation	[62,63]
Pest and disease management	Predict pest and disease outbreaks	[58,64,65]
Supply chain optimization	Monitoring crop growth, forecasting harvest times, coordinating logistics, minimizing post-harvest losses, and ensuring timely market delivery	[16,23,54,[66], [67], [68]]
Yield prediction and management	Allowing farmers to make better decisions about harvest timing, storage, and market planning	[67,[69], [70], [71]]
Climate impact analysis	Evaluating the effects of climate change on crop growth and health	[29]
Resource optimization	Integrating data on soil health, weather, and crop requirements	[57,72]
Sustainability and compliance	Adhering to sustainability standards, maintaining farming records, and marketability	[30,51,67,72]

### 3.3 General architecture of digital twin implementation

Digital twins are a system that utilizes sensors and other data sources to optimize crop management. These devices collect data from various sources, such as soil moisture, temperature, humidity, and environmental variables [8,73]. The data is then integrated into a digital twin model using algorithms and analytics to create a virtual representation of the crop. This data is then used to make informed decisions on crop management, such as optimizing resource usage and predicting crop yields. By analyzing data from weather forecasts, soil moisture, and crop growth, digital twins can recommend the ideal time to apply fertilizers or harvest crops.

The design of the digital twin consists of three layers: the physical world, communication protocol, and cyber world [8]. Sensors are attached to the smart farm field to collect data on temperature, humidity, air velocity, light conditions, ventilation, and crop growth. Additionally, innovations, such as satellite-based remote sensing, are contributing to the development of more accurate digital twins by providing high-resolution imagery of the farm.

**Fig. 6:**
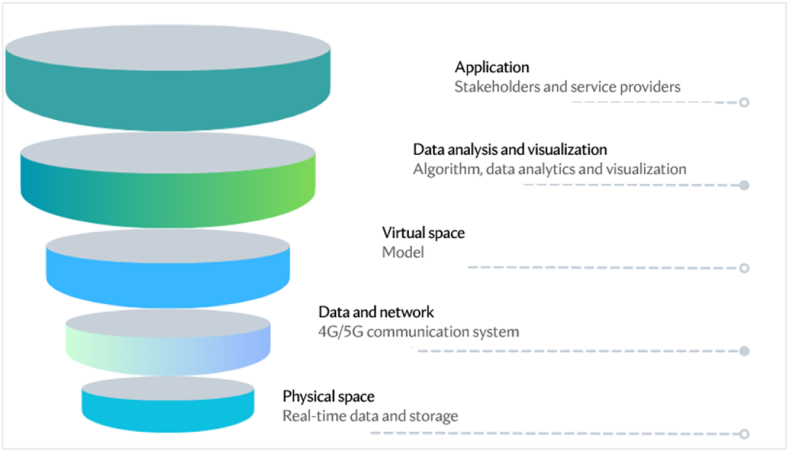
Digital twin architecture for crop management.

Fig. 6 depicts the smart farming digital twin, which consists of five layers: physical space, data, network, digital space, data analysis, and visualization, as well as an application layer with service providers and stakeholder alerts. Environmental sensors, camera sensors, and chemical sensors are used to collect agricultural data. The storage layer stores current and historical data for productivity optimization. Thus, historical data on crop yields, weather patterns, and soil conditions can help predict future crop performance and optimize resource allocation [74]. The data analysis and visualization layer hosts rules and knowledge to choose among crop treatments and climate control strategies to optimize farm productivity. The model is powered by AI and ML algorithms to increase visibility and provide insights to farmers and stakeholders. Farmers and other stakeholders can get updates regarding the farm's state through the application layer. For example, a farmer might receive an alert on their mobile phone indicating the best time for irrigation based on real-time data and forecasted weather conditions [25].

### 3.4 Challenges and prospects

#### 3.4.1 Implementation challenges

Digital twin technology in crop management faces several challenges, including poor data quality, data integration issues, the complexity of the model, the cost of technology, cyber security, and data privacy [[75], [76], [77]]. Widespread adoption is limited due to a fragmented modeling landscape, difficulties in capturing all relevant processes with models, and IT infrastructure [13]. Digital twin models rely on high-quality data, which can be difficult to obtain in agriculture due to variability in environmental conditions and other factors. Managing data from diverse sources can be challenging due to the varied formats and standards used by different sensors and platforms. Moreover, the agriculture industry may face challenges in developing accurate digital twin models due to the complexity and time-consuming nature of data analytics, modeling, and simulation [78].

Cybersecurity is a significant concern in implementing digital twin technology in agriculture, particularly for smaller farmers or those in developing countries, as it requires ensuring data security and protection from unauthorized access [79]. Collection and utilization of data through digital twin technology can pose privacy concerns, particularly if the data contains personal data or sensitive information about farmers or their land. Yet another problem for implementing digital twins in outdoor agricultural fields is the need for dynamically changing field conditions, as well as the complexity of replicating living things [16]. Existing applications are generally focused on basic monitoring features or fine-granular object virtualization, with lower granularity management being expensive and missing integrated software options [10]. Moreover, they face issues such as a lack of internet connectivity in rural areas, concerns about the physical security of equipment, and difficulty maintaining infrastructure due to geographic isolation. The high cost of investment and a lack of knowledge about crop varieties and farming conditions also hinder adoption.

Understanding biological, chemical, and physical processes in plants and soils during mode development is essential for agriculture, making it difficult to model dynamic systems. Similarly, satellite-collected data accuracy is heavily influenced by weather conditions causing wrong information. Further, synchronization between physical and virtual twins is another challenge [80].

#### 3.4.2 Prospects

Digital twin technology is poised to revolutionize the agricultural industry by enhancing crop life cycles, automating processing and production, and modeling animal living conditions, nutrition requirements, and variety breeding [81]. As this technology advances, it offers numerous benefits, including increased efficiency, lower costs, improved crop quality, and increased sustainability [82,83]. Farmers can use digital twins to optimize crop management operations such as planting, fertilizing, and irrigation, making data-driven decisions that boost yields and decrease waste.

Digital twins can also assist farmers in reducing their environmental impact by optimizing resource utilization, such as water and fertilizer. By using data to make more informed decisions, farmers can reduce waste and make better use of resources. Additionally, digital twins can enable agricultural businesses to meet customer demand, allowing growers to monitor crop growth and development, resulting in increased yields and higher-quality food.

As digital twins become more deeply integrated into agricultural practices, the future holds immense potential for precision agriculture optimization, predictive analytics, resource optimization, remote monitoring, and decision support systems. By developing virtual models of entire agricultural operations, including crops, fields, machinery [84], and environmental conditions, farmers can gain valuable insights into crop growth [85], soil moisture levels, nutrient requirements, pest and disease outbreaks, and other factors [86]. This leads to increased efficiency, lower costs, and higher crop yields [87].

Predictive analytics can also revolutionize agriculture by simulating different scenarios and analyzing historical data, enabling farmers to identify inefficiencies in resource usage such as water, energy, and fertilizers [2,3,77]. This enables targeted strategies for reducing waste and environmental impact, leading to more sustainable farming practices and the conservation of valuable resources.

Digital twins are revolutionizing agricultural operations by enabling remote monitoring, automation, and early detection of crop conditions. This reduces labor requirements and improves farmers' operations. With advancements in technology and agricultural virtualization methods, digital twins could potentially recreate real-life environments in cyberspace, paving the way for a more resilient and food-secure future. This technology could revolutionize cultivation, management, and optimization within agriculture.

## 4 Conclusions

This article discusses the advancements in crop monitoring digital twin applications, examining various modeling approaches used in crop management. The study also highlights the role of digital twins in crop management, the architecture for implementation, challenges, and prospects.

Despite its early development, digital twins could be a significant step in agriculture's digital transformation, providing a better return on investment, improving farmers' decision-making, making business owners more cost-efficient, and improving agricultural output, economic growth, and sustainability in the face of climate change. However, the use of digital twins in crop management is still in its early stages, and long-term analyses of its performance and sustainability are required to fully understand its potential.

The review contributes to Industry 4.0 and 5.0 by providing insights into technology integration, sustainability, and resilience in the agricultural sector. Furthermore, this work makes an important theoretical contribution by expanding the scope of digital twin technology as a revolutionary tool for crop management. It also introduces frameworks for combining diverse modeling methodologies and predictive analytics, setting a foundation for future research in precision agriculture.

Future research should focus on establishing standardized methodologies, comparing digital twin approaches with traditional practices, examining the impact of digital twins over multiple growing seasons or agricultural cycles, and exploring the perspectives, needs, and challenges faced by end-users in integrating digital twin technology into existing practices.

## Data availability statement

All the data used in this research are included in the article.

## Funding information

No funding resource could be reported for this publication.

## Declaration of competing interest

The authors declare that they have no known competing financial interests or personal relationships that could have appeared to influence the work reported in this paper.
